# Public Health Messaging and Strategies to Promote “SWIFT” Lung Cancer Detection: a Qualitative Study Among High-Risk Individuals

**DOI:** 10.1007/s13187-020-01916-w

**Published:** 2020-10-31

**Authors:** Mohamad M. Saab, Caroline Kilty, Brendan Noonan, Serena FitzGerald, Abigail Collins, Áine Lyng, Una Kennedy, Josephine Hegarty

**Affiliations:** 1 Catherine McAuley School of Nursing and Midwifery, College of Medicine and Health, University College Cork, College Road, T12 AK54, Cork, Ireland; 2 National Cancer Control Programme, Health Service Executive, Dublin, Ireland

**Keywords:** Awareness, Early detection, Focus group, Health disparity, Health promotion, Lung cancer, Qualitative research

## Abstract

Lung cancer (LC) is the most common cancer and the leading cause of cancer mortality globally. A positive association between LC incidence and socioeconomic deprivation exists. High-risk individuals are less likely to be aware of LC and to correctly appraise LC symptoms and seek medical help accordingly. This qualitative study explored strategies to promote early detection of LC among at-risk individuals living in high-incidence areas in Ireland. Five semi-structured focus groups were conducted with 46 individuals. Data were collected face-to-face in community centres and organisations in high-incidence areas in two Irish counties and analysed using inductive qualitative content analysis. Participants believed that there was insufficient information regarding LC and recommended promoting LC awareness at a young rather than old age. They favoured public health messages that are **S**imple, clear, and honest; **W**orded positively; **I**ncorporating a shock element; **F**eaturing a celebrity, healthcare professional, or survivor; and **T**argeted (**SWIFT**). Most participants reported becoming immune to messages on cigarette packaging and recommended using a combination of broadcast and print media within national government-run campaigns to promote LC awareness and early detection. Study findings suggest that promoting LC awareness, help-seeking, early presentation, and diagnosis can be achieved by developing and testing targeted interventions. Promoting LC awareness requires a multi-sectoral policy network, or a whole systems approach. Such approaches ought to consider the multifactorial drivers of LC risk behaviours; involve coordinated, collective actions across various stakeholders; operate across multiple agencies; and take a life course perspective.

**Supplementary Information:**

The online version contains supplementary material available at 10.1007/s13187-020-01916-w.

## Introduction

Lung cancer (LC) is the most common cancer and the principal cause of cancer mortality globally, with 2.09 million cases and 1.76 million deaths in 2018 alone [1]. In Ireland, LC ranks first among invasive cancer deaths, with almost 60% of cases diagnosed in late stages [2]. The median age group at diagnosis and death is 70 to 74 years and the 5-year relative survival rate for LC is 17.9%. In total, 1,407 males and 1,157 females are diagnosed with LC and 1,069 males and 785 females die from it annually in Ireland [2].

A positive association between LC incidence and socioeconomic deprivation exists. An analysis of 12 case-control studies from Europe and Canada with 17,021 LC cases and 20,885 controls found that, after adjusting for smoking, low socioeconomic status was significantly associated with a higher risk of LC [3]. Similarly, a national report on cancer inequalities in Ireland found a trend of increasing LC incidence with increasing deprivation, with approximately 60% higher incidence among the “most” compared with the “least” deprived populations [4]. In addition, urban dwellers in high deprivation areas were found to have a significantly higher LC incidence, were more likely (6%) to be diagnosed at late stages, and were less likely (− 21%) to undergo surgery for LC in comparison to rural dwellers [5]. Of note, surgery is considered to be the optimal treatment strategy for localised LC, whereas chemotherapy has been shown to reduce mortality in advanced LC [4].

High-risk individuals are less likely to be aware of LC and to correctly appraise LC symptoms and seek medical help accordingly [6]. This can have detrimental effects on early diagnosis [1] and potentially lead to lower eligibility for LC treatments linked to less invasiveness, fewer sequelae, and increased survival time [7]. Thus, raising LC awareness and promoting early help-seeking and diagnosis among vulnerable populations are key. This is in line with recommendation seven of Ireland’s National Cancer Strategy 2017–2026 which calls for developing “a rolling programme of targeted multi-media based public awareness and education campaigns, aimed at the early detection of specific cancers and with particular focus on at-risk populations” (p.134) [8].

The purpose of this qualitative study is to explore strategies to promote early detection of LC among at-risk individuals living in high-incidence areas in Ireland. Ultimately, study findings will inform the development of a national intervention to enhance LC awareness, help-seeking, and early detection.

## Methods

### Study Design

This qualitative study draws from the general tenets of the naturalistic inquiry with no prior commitment to existing theories, philosophies, or epistemological stances [9]. To enhance research quality and maintain an audit trail, this study is reported using the Standards for Reporting Qualitative Research checklist [10].

### Participants

Convenience sampling was used to recruit participants from community centres and organisations in Irish counties Carlow and Dublin which have the highest incidence of LC [2]. Individuals aged 50 years or more and who have high LC risk (e.g. smoking, family history of LC, and/or exposure to occupational/environment hazards) were eligible for inclusion. LC survivors were not eligible for inclusion; however, two LC survivors sat on one of the focus groups. Data from these individuals were included in this study for ethical and pragmatic reasons.

### Data Collection

Before each focus group, study participants signed informed consent and completed a sociodemographic questionnaire with 13 questions on age; gender; nationality; marital status; education; employment; occupation; health insurance; address; living arrangement; smoking status; cigarettes smoked per day; and years smoking [11, 12].

Focus group rules were agreed beforehand including the importance of speaking one at a time and capturing everyone’s views. Authenticity was enhanced by using icebreakers, providing refreshments, and using name tags for participants and interviewers. Five audio-recorded focus groups lasting on average, 82 minutes were conducted face-to-face in two community organisations and one community centre in February 2020. Each focus group was facilitated by two experienced interviewers who had no previous relationship with the participants. A semi-structured focus group guide designed elsewhere [11] explored participants’ (i) views regarding raising LC awareness and promoting early detection, (ii) recollection of previous interventions/campaigns, (iii) personal interest in learning more about LC and (iv) views regarding previous LC awareness campaigns (see supplementary Table S1 for the semi-structured qualitative interview guide).

### Data Analysis

Focus groups were transcribed verbatim. Data analysis and collection were concurrent. Reflexivity serves as a process of continual self-evaluation and dialogue between researchers while actively acknowledging and recognising their influence on the study process and outcomes [13]. In the current study, interviewers’ (MMS, CK, BN, SF) reflexivity was addressed by keeping audio-recoded memos pertaining to focus group process, content, and future iterations immediately after each focus group. A summary of these memos was then prepared by the lead author (MMS) and shared with the whole research team. Therefore, data from this study were analysed iteratively whereby discussions in earlier focus groups and interviewers’ reflexive memos informed the content of future focus groups.

Focus groups were analysed using inductive qualitative content analysis [14]. Excerpts were condensed into codes by four authors (MMS, CK, BN, SF). Codes were transferred to a coding sheet and sub-categories were created to collate and collapse similar codes (see supplementary Table S2 for a sample coding sheet). Similar sub-categories were then grouped, and broader categories were generated inductively. Sociodemographic data were inputted into SPSS and analysed using descriptive statistics.

### Ethical Considerations

This study received ethical approval from the Social Research Ethics Committee at University College Cork. All participants provided written informed consent. An information sheet was provided to participants explaining the risks and benefits from their participation and providing contact details of free cancer support services.

## Results

### Participant Characteristics

Five focus groups were conducted with 46 participants, with 5 to 11 participants per focus group. Participants’ mean age was 69.6 years for males (range: 51–83 years) and 67.9 years for females (range: 51–90 years). The majority of participants were female (65.2%), Irish (97.8%), married (37%) or widowed (28.3%), and have completed either primary (41.3%) or secondary (41.3%) education. Most participants (58.7%) were retired, medical card holders (82.6%) (i.e. eligible for free medical care) and lived either in their own homes (47.8%) or in houses rented from a local authority (37%). Half of the participants reported being either former smokers (37%) or having never smoked (13%). Current smokers (50%), including social smokers, smoked between 10 and 40 cigarettes per day for 20 to 75 years (Table 1).

**Table 1:** Participant characteristics^a^

Characteristic		n (%)
Age (years)	Range	51–90
	Mean (standard deviation)	68.5 (10.5)
Gender	Female	30 (65.2)
	Male	16 (34.8)
Nationality	Irish	45 (97.8)
	English	1 (2.2)
Marital status	Married	17 (37)
	Widowed	13 (28.3)
	Single	6 (13)
	Divorced	5 (10.9)
	Separated	3 (6.5)
	Partnered	2 (4.3)
Education	Primary	19 (41.3)
	Secondary	19 (41.3)
	University/college	8 (17.4)
Employment	Retired	27 (58.7)
	Employed (part-time)	9 (19.6)
	Disabled	3 (6.5)
	Unemployed	3 (6.5)
	Homemaker	2 (4.3)
	Employed (full-time)	1 (2.2)
	Volunteer	1 (2.2)
Occupation^b^	Cleaner	2 (20)
	Day-care assistant	2 (20)
	Maintenance	2 (20)
	Other	4 (40)
Health insurance	Medical card	40 (87)
	Private insurance	2 (4.3)
	Medical card and insurance	2 (4.3)
	General practitioner card and insurance	1 (2.2)
	Medical and general practitioner cards	1 (2.2)
Address	Urban	30 (82.6)
	Rural	8 (17.4)
Living arrangement	Own	22 (47.8)
	Rent from local authority	17 (37)
	Mortgage	3 (6.5)
	Living with family/friends	2 (4.3)
	Rent privately	2 (4.3)
Smoking status	Smoker	22 (47.8)
	Ex-smoker	17 (37)
	Never smoked	6 (13)
	Social smoker	1 (2.2)
Cigarettes per day^c^	Range	10–40
	Mean (standard deviation)	18.4 (7.2)
Years smoking^c^	Range	20–75
	Mean (standard deviation)	44.4 (13.5)

^a^*n* = 46 unless otherwise indicated

^b^*n* = 10 participants who were employed

^c^*n* = 22 participants who were current smokers

Three categories were constructed from the data: (i) information needs, (ii) suggestions for effective public health messaging, and (iii) information platforms and preferred learning strategies (Table 2).

**Table 2:** Study findings with participants’ verbatims

Information needs	
“There isn’t enough information out there”	“There isn’t enough information out there to tell us about what’s this and what’s that…I think there should be more information…and I truthfully say there’s never been anything like that [focus group discussion] started in this country.” (FG3)
Need for information on early signs, symptoms, and risk	“What you’re actually looking for, not the changes in your voice or you’re coughing. What is it you’re actually looking for the early stages of cancer because I don’t know any of them?...I think if you were made more aware of, what kind of coughs you’re looking for, it would make you think more…” (FG1)
“It’s too late for us… target young people”	“That’s too late for us…I can’t understand why people are targeting older people. Why aren’t they targeting young people starting off?...” (FG2) “For us, our days are coming to a, well, the near end…like me, I started taking instalments out for my funeral. That’s my age, but it’s to get the lung cancer thing at an early age. We’ll say show somebody like yourselves [interviewers] or younger…somebody who will look at that. There’s no point in showing it to me.” (FG3)
Suggestions for effective public health messaging **(SWIFT)**	
**S**imple, clear, and honest	“Clarity, and simplicity in messages targeted to help them [at-risk individuals] become more aware of symptoms and what they need to do in case they felt a symptom…If you went to a doctor and you said you smoked X amount a day and then the doctor literally said he did some tests on you and it’s not looking good and if you don’t quit smoking in the next seven to 10 days, whatever means you need to quit, you won’t be around in six months’ time. Well, guess what, you’re going to go out of there with food for thought…that would make a hell of a lot of people quit thinking oh, I’ve got six months.” (FG1)
**W**orded positively	“I read a leaflet and they said how much money you’d save and I’m sorry, but that kind of did it…a family holiday and of course you feel like the worst mother in the world then because you can’t bring your children on holidays because you’re smoking.” (FG1) “There was a [message] saying the day after you give up smoking, your lungs start to recover.” (FG4)
**I**ncorporating a shock element	“If you look at the charity advertisements for Africa and you see a lady with a baby in her hand and they’re looking for money to help, to sort this problem out, if you have the same thing with a new-born baby and a mother holding her and say this baby smokes 60 cigarettes a day, how long is this baby going to live? Just something…a shock.” (FG5)
**F**eaturing a celebrity, healthcare professional, or survivor	“They should really give a good ad, some well-known boy or girl to say a few words. Some young celebrity that is well-known and the caption underneath him or better still, if the camera was on him and the mic near him, that you could hear him saying the words.” (FG3)
**T**argeted	“You don’t need these pictures [on cigarette packaging]. God forgive me for saying this, but I don’t need to be told smoking will make me impotent. I’ve three kids and I smoked all my life. But keep it simple and get people, aim it at a younger age group who will take it on board. Like aim it at maybe a slightly different type of leaflet for people in our age group. The same information, but a different format.” (FG3)
Information platforms and preferred learning strategies	
Sources of previous information	“In the sixties, I remember in [shop name] there was this Conquer Cancer campaign. They only discovered that…they proved that smoking caused cancer. Up to then, there wasn’t because they would be no…all the labels then on the cigarettes, they had to have a label to say that smoking causes cancer, but before that, it was never proven. So, when it was proved, they had this big campaign going on the television, on the radio, things like that, Conquer Cancer, stop smoking.” (FG5)
Multimodal campaigns	“I think myself a video should be done, a recording of some sort. You have a smoker that has cancer and then you have a survivor that had cancer and gave up the fags [cigarettes]. And it would be good awareness of the difference of both and let the two sides tell the story. Why this person, even though they have cancer, why they can’t give up the fags or why they feel it’s not necessary to give them up or why they started smoking. The other person, why they gave them up, how long they were smoking before and what possessed them to start smoking in the first place…” (FG2) “Why don’t you have an advertisement and you had say the likes of us and we have a room and you have one person just cough and then you progress to three weeks later? And then you send the message out. That cough was cancer.” (FG4) “Department of Education, should get the leaflets to all schools…Department of Health or the HSE [Health Service Executive] should have a leaflet and give it out to everybody or have it up in the chapels, churches, chemist, doctors, dentists…anywhere where [they] have to sit and wait for a while because [they] will have to pick it [leaflet] up.” (FG5)
Discreet diagnostic services and face-to-face support	“The mobile, BreastCheck [national breast cancer screening service] unit…if they had something like that where it’s not in the middle of town…or in a pharmacy…somewhere that people don’t see you going in and out…that you maybe could go and get an x-ray just for that…because everybody is their own individual person and the majority of people like to keep their business to themselves…so they don’t want to run into people they know and have them gossip behind their back.” (FG1) “Have a one-to-one support person that’s willing, that has given up the fags [cigarettes]…there’s no point in having somebody that never put a fag into their mouth to support you because they don’t know what you’re going through. You need somebody there that’s after being smoking, after giving them up, went through the mill, knows what you’re going through and is able to help you out there and then, you know.” (FG2)
Potentially ineffective strategies	“But do you see, if there’s a packet of fags [cigarettes] sitting and you can see all that stuff or you’re seeing lungs or something, I’ll turn that packet over…I don’t see that anymore....you just don’t see it….you’re immune to it.” (FG1) “Putting it [message] on the back of a cigarette packet is too late. You’re already going to smoke, you know…there’s no point in showing me a picture of blackened lungs on a packet of cigarettes that I’m going to take one out of.” (FG3)
Views regarding pre-existing campaigns	“Be Clear on Cancer” (England): “Easy to read, easy to understand…has a good lot of awareness. It is the signs and symptoms and it gets you thinking. Do you have a recurring cough and it wasn’t going away?...it is straightforward.” (FG1) “You have scenarios…it [leaflet] raised my awareness of it [lung cancer]. If I picked that up now and that, if I saw them in the doctors…I’d be more drawn to this because of the…[personal stories].” (FG2) “Leaflet is cold and clinical…there’s nothing in that [leaflet] now that would make me look twice…been coughing for three weeks [message] is not applicable because [he has] been coughing for 55 years.” (FG3) “Leaflet is a bit heavy…not really inclined to read all that…nobody’s going to pick that [leaflet] up and read it.” (FG4) “[The slogan ‘Be Clear on Cancer’] is boring…and people wouldn’t be bothered reading it.” (FG5). “Get Checked Early” (Scotland): “If that [leaflet] came in my door, I would read it…I think it’s [leaflet] very good because personally, myself, I haven’t seen anything like this written down to say lung cancer doesn’t have to be game over…I always thought once you had cancer, you had cancer, especially lung cancer.” (FG1) “It [leaflet] has Alex Ferguson [celebrity] on it…especially for young lads because they look up to him…it might be too late for me, but for my sons, they’d look up to him big time.” (FG2) “I find [the slogan ‘Don’t Get Scared Get Checked’] comforting…it doesn’t give the impression you have lung cancer you’re going to die.” (FG3) “Giving a list of bullet points [favourable]: if you have two or more of these symptoms, get it checked out. It could be lung cancer.” (FG5)

### Information Needs

Focus group (FG) participants highlighted a knowledge gap about LC nationally and believed that “there isn’t enough information out there” (FG3) and that “more publicity on lung cancer” (FG1) was needed. Encouragingly and interestingly, several participants attended focus groups to inquire about LC and learn more about its risk factors, signs, and symptoms such as the significance of “coughing up blood” (FG3) and the “relationship between LC and COPD [chronic obstructive pulmonary disease]” (FG5).

In terms of raising LC awareness and promoting early detection, some participants stated that, given their age and smoking history, it was “too late” (FG2) for them and that their “days are coming to a near end” and the “harm is done” (FG3). Alternatively, many recommended that smoking cessation and LC awareness should start at a younger age and should “hit the schools” (FG3), rather than targeting people who are older than 50 years.

### Suggestions for Effective Public Health Messaging (SWIFT)

While exploring public health messaging to promote LC awareness, help-seeking, and early detection, participants favoured messages that are **S**imple, clear, and honest; **W**orded positively; **I**ncorporating a shock element; **F**eaturing a celebrity, healthcare professional, or survivor; and **T**argeted (acronym: **SWIFT**). Generally, participants favoured “clear and understandable” (FG3) messages to raise awareness of the early signs and symptoms of LC and to enable them to know “what [they] have to do” (FG3) and “when to seek help” (FG1). Participants also wanted clear information about “specific symptoms directed towards the lungs” (FG1). For many, honesty was in the form of a doctor linking the number of cigarettes smoked per day to life expectancy. This was perceived as potentially effective in promoting smoking cessation and reducing LC incidence as a result.

While some participants recommended using scare tactics to raise awareness, the majority was against “putting fear into people” and said that “comfort might work too” (FG3). For example, FG1 participants did not favour the use of graphic images and negative messages like “if you smoke, you die” on cigarette packaging. Instead, they recommended having a list of sources of help for LC symptoms and bullet points with LC alarm symptoms. Others recalled reading about the money saved following smoking cessation and gave the example of a mother who could not afford to take her children on a holiday because she smoked. In keeping with positive messaging, FG4 participants iterated the importance of messages like “the day after you give up smoking, your lungs start to recover” in helping with smoking cessation and reducing the impact of LC as a result.

Creating “panic and shock rather than fear” (FG5) was preferred by most participants. For many, the element of surprise was mostly targeted towards smoking cessation rather than LC per se. For instance, several participants in FG5 recommended the use of “shockvertising” to get the message across such as showing a video of a mother giving her baby cigarettes, a teenager who had LC, and an attractive man whose partner refused to kiss because he smelled “like a dirty ashtray” (FG5).

Putting a face to an advertisement was favoured by many participants. Some recommended using celebrities, while others disagreed and suggested using reliable sources of information such as healthcare professionals or people who had first-hand experience with LC.

Participants stressed the importance of tailoring public health messages and targeting various generations and age groups differently. For example, one participant said: “I don’t need to be told smoking will make me impotent. I’ve three kids and I smoked all my life!” Instead, he favoured information “geared towards [his] age group” so that he would take “the information on board” (FG3). Another said that “what we [his generation] look at is different what the young people look at” (FG3).

### Information Platforms and Preferred Learning Strategies

While exploring sources of pre-existing knowledge of LC, very few participants were able to recall previous sources of cancer information including a campaign in the 1960s titled “Conquer Cancer” (FG5), a newspaper segment about a celebrity who “quit smoking at the age of 76 years” (FG2), and a video on cancer awareness in the workplace.

Participants’ preferred strategies to learn more about LC were gleaned iteratively, whereby learning strategies identified in one focus group were presented and discussed in subsequent focus groups. Overall, participants favoured platforms where information on LC is acquired passively rather than them actively seeking the information. As a result, several broadcast (e.g. videos and television) and print (e.g. leaflets, posters, and billboards) media were identified. A combination of both media into wider national government-led campaigns was favoured by most if not all participants. In terms of frequency of delivery, campaigns were recommended to “run all the time” (FG4), during public holidays, or during “an awareness day all over Ireland…one day of the year” (FG3).

Many participants had a clear vision as to what they would like to see in the campaign’s advertisement. They recommended advertisements that feature two distinct personalities telling their stories with smoking and LC such as a smoker with a non-smoker; an older person with a teenager; and a smoker who has cancer with a cancer survivor who quit smoking. In terms of content, participants preferred having “a list of bullet points: if you have two or more of these symptoms, get it checked out. It could be lung cancer. And put the word ‘lung cancer’ on the television” (FG5). Others favoured having details of where people can go and seek help for symptoms of concern. This was recommended by a participant in FG4 who recalled watching a television advertisement about free services that offer cardiac screening.

Participants in all focus groups recommended using leaflets, posters, and billboards “everywhere” including buses and bus stop; trains; trams; places of worship; chemists; doctors’ surgeries; dentists; libraries; restaurants; public toilets; public houses; on social media/phones; close to schools; and by post. Facts and information published by doctors and researchers were also suggested for dissemination “in every county in Ireland” (FG2). Participants also believed that campaigns must be driven by the government and that “the government should send out leaflets everywhere and say: ‘this is what we’re going to do to try and prevent it [LC]’” (FG3).

Fewer participants recommended face-to-face support and discreet diagnostic services. One participant in FG1 recommended “a support group… you don’t feel alone.” This was echoed by a participant in FG2 who endorsed support by ex-smokers who “know what you’re going through and are able to help you out there and then” (FG2). For participants in FG1 and FG2, discretion and privacy were key to promoting LC early detection. Many “do not want to run into people they know and have them gossip behind their back” (FG1) and recommended mobile units where they could get chest X-rays in private.

In contrast, participants reflected on strategies that they believed were ineffective in promoting LC awareness and early detection. Many participants stated becoming “immune” to graphic messages on cigarette packaging, while others mentioned that they were “gone with the fairies when it comes to technology” and favoured “a meeting type setting…face-to-face…for [their] age” (FG3).

Before the end of each focus group, participants were provided with posters and leaflets from two prominent LC awareness campaigns run by the National Health Service (NHS) in England (Be Clear on Cancer) [15], and Scotland (Get Checked Early) [16]. Participants took 10 minutes to read through each campaign and reflect on content and layout.

The first campaign (Be Clear on Cancer) features a doctor explaining what symptoms to look for and contains personal stories from LC survivors. The second campaign (Get Checked Early) features Sir Alex Ferguson, a Scottish former football manager and player who lost both parents to LC. This campaign had bullet points with key signs and symptoms of early LC and used the slogan “Don’t Get Scared Get Checked.”

Overall, the “Be Clear on Cancer” campaign was perceived as “informative” (FG5) and “easy to read, easy to understand” (FG1). Participants appreciated positive messages such as: “it’s not all smokers…one in eight people get lung cancer that are not smokers” (FG5). However, some participants perceived the leaflet as “a bit heavy” and were not “really inclined to read all that” (FG4). Moreover, participants in FG3 thought that the leaflet was “cold” and “clinical.” A participant in FG4 said that the campaign message about the importance of help-seeking for a cough lasting for three weeks or more would make people “look quicker…because the coughing is the bit that gets you.” He added: “but there’s nothing in that [leaflet] now that would make me look twice.” Another participant perceived the message “been coughing for three weeks?” as not applicable to him “because [he has] been coughing for 55 years!” (FG3). The leaflet included personal stories from LC survivors. These were favoured by some and perceived as ineffective by others.

As for the NHS Scotland campaign (Get Checked Early), most participants appreciated the slogan “Don’t Get Scared Get Checked” and found the statement “lung cancer doesn’t have to be game over” positive, reassuring, and “comforting” (FG3). However, the message “lung cancer isn’t what it used to be” did not appeal to one participant who said: “I’d move off of that. It is [what it used to be], and it’s not going to change until everybody get up and do something about it” (FG3).

Most participants were in favour of using Sir Alex Ferguson as the face of the campaign since “the chances are nine out of 10 people will know who he is” (FG3) and “young lads look up to him” (FG2) and “take heed of him” (FG5). Participants were also drawn to the colours and fonts used in the Scottish campaign and favoured the list of symptoms that were bullet pointed in the leaflet. It was suggested, however, that the bulleted list of symptoms, the picture of the celebrity, and the slogan “Don’t Get Scared Get Checked” should be displayed on the leaflet cover which can then be used as a poster, banner, and/or billboard.

## Discussion

Findings from this study suggest that individuals have varied preferences regarding learning more about LC. While most participants highlighted that they needed more information about LC, there were several conflicting perspectives articulated. The convergence of factors that stigmatise smoking, the perceived inability to ward off LC, coupled with fatalistic beliefs relating to LC and the notion that it was too late for the participants, and it was best to focus on young people are some of the potential difficulties faced by those involved in developing health-promoting interventions.

Participants tended to deflect the responsibility for raising awareness to others, noting the government’s responsibility to inform them about LC and to support their help-seeking actions. In addition, most participants noted that they get information about cancer through coming across it passively in their daily lives, instead of actively seeking this information. As a result, they recommended having the information available to them in the form of television shows, videos, billboard advertisements, posters, and leaflets that they can access in public spheres including bus stops, churches, pharmacies, clinics, and libraries among others. Similarly, Drummond et al. [12] found that most participants passively acquired cancer-specific information and men with low health literacy were less likely to obtain cancer information both passively and actively.

Focus group participants described the message characteristics they favoured (i.e. SWIFT) and noted the importance of multimodal campaigns. In this regard, a wide variety of approaches can be used to increase LC awareness including generic mass and small media awareness campaigns [17, 18]; targeted, intensive community-based behaviour change interventions [19]; and making each health consultation count [20]. More targeted interventions, which draw upon pre-existing social networks and influences and existing healthcare provider relationships, tend to be more successful at improving cancer awareness in high-risk disadvantaged populations [21]. In addition, interventions that draw from or are underpinned by theory are more likely to help raise cancer awareness [22].

Participants noted that pre-existing messages around smoking and LC seemed to contribute to their fatalistic views due to a focus on death rather than help-seeking, treatment, and/or survival. Thus, participants noted the importance of simple, clear, honest, and positively worded health messages. Health-promoting interventions need to empower individuals to seek help early, while informing them about the risks and symptoms of LC and concurrently dispelling negative fatalistic and stigmatising beliefs about LC and treatment outcomes [6]. The example of cigarette packaging perpetuating these fatalistic beliefs was provided by many participants.

Some participants highlighted that they became immune to health-promoting messages in the long term, thus the need to reinvigorate the message and, at times, use a shock element. Indeed, shock advertising or “shockvertising” is used widely in heath awareness campaigns. While this type of advertising may be effective, the public tends to grow immune to it [23]. This was the case in the present study whereby many participants reported being unaffected by graphic pictures and messages used on cigarette packaging.

In terms of existing awareness campaigns run by the NHS in England (Be Clear on Cancer) [15] and Scotland (Get Checked Early) [16], participants particularly liked the acknowledgement of their potential fears and the positivity and clarity of the messages in both campaigns. In fact, studies evaluating the effect of the “Be Clear on Cancer” campaign in the UK found a significant increase in LC awareness and help-seeking behaviours and a significant decrease in the number of patients diagnosed with late-stage LC [17].

Improving LC awareness necessitates multiple intervention strategies, which requires a multi-sectoral policy network [24], or a whole systems approach [25]. Such approaches ought to consider the multifactorial drivers of smoking and LC risk behaviours; involve coordinated, collective actions across a range of stakeholders; operate across all levels of government working at multiple levels among multiple agencies; and take a life course perspective.

Promoting population-level LC awareness, particularly in at-risk groups is a complex and difficult task which requires a coordinated and sustained effort by government and policy makers. Given that over 1,000 European citizens die from LC and 30 to 50% of all cancer cases are preventable through increasing awareness, reducing exposure to risk factors, and addressing modifiable lifestyle factors [26], researchers ought to develop an agreed understanding of what a whole systems approach in relation to promoting LC awareness means. Given that multimodal and multiagency interventions are needed to increase LC awareness across the lifespan, it is important that evaluation research seeks to understand the mechanisms through which health-promoting interventions improve LC awareness, help-seeking behaviours, and eventually clinical outcomes (e.g. earlier detection) in different population groups.

### Recommendations for Future Research

In the current study, it was hard unifying views which would inherently be ununified and dissimilar. However, the iterative process used during data collection and analysis helped clarify the dos and don’ts of public health campaigns targeted at raising awareness and help-seeking, and potentially promoting early detection of LC among this high-risk cohort. Overall, study participants favoured government-led multimodal educational campaigns or rolling programmes targeted at promoting awareness, help-seeking, and early detection of LC among at-risk individuals, while reporting becoming immune to pre-existing strategies such as messages on cigarette packaging.

Current study findings stress the need to empower at-risk individuals to seek help early. This can be achieved through delivering information on LC that participants thought were important such as LC risks, signs, and symptoms which warrant seeking help from a GP, while dissipating misconceptions and fatalistic misbeliefs around LC.

Accounts from study participants led to the development of the acronym **SWIFT** which can be used to design and deliver effective public health messages on LC that are **S**imple, clear, and honest; **W**orded positively without using scare tactics; **I**ncorporating a shock rather than a fear element; **F**eaturing a public figure such as a celebrity, healthcare professional, or LC survivor; and **T**argeted towards high-risk individuals rather than being a “one size fits all”-type intervention. Indeed, complex health interventions work best when tailored to certain situations rather than being entirely standardised [27].

Given the sociodemographic characteristics of the target population and the varying levels of literacy and health literacy, information that is accessible to individuals in their day-to-day life ought to be considered. Indeed, study participants recommended delivering LC information using broadcast (e.g. videos and television) and print (e.g. leaflets, posters, and billboards) media that are made available on public transport; places of worship; chemists; doctors’ clinics; libraries; restaurants; public toilets; public houses; online; close to schools; and/or by post.

Frameworks for intervention development such as the Medical Research Council Framework ought to be considered to ensure a rigorous process of intervention development, feasibility/pilot testing, evaluation, and implementation [27]. Studies with a theoretical underpinning are more likely to produce the desired effect [22]; therefore, behaviour change models can be used to develop and test future interventions. Examples include but are not limited to the Health Belief Model [28] and the Theory of Planned Behaviour [29] or more recent models and frameworks such as the Behaviour Change Wheel [30] and the Preconscious Awareness to Action Framework [22]. In terms of outcome measures, intervention effectiveness ought to be evaluated by means of self-report (e.g. awareness and help-seeking) and/or objectively (e.g. number of new referrals for suspected LC; physician-prescribed chest X-rays/chest computer tomography [CT] scans; number of new LC cases; LC stage at diagnosis; treatments received; and survival rates).

### Limitations

While several measures were taken to enhance trustworthiness, it is worth considering the limitations of this study. Only participants who volunteered to participate were interviewed, which increases the risk of self-selection bias. Including two participants with a history of LC could have biased responses from other participants. However, this served as a space for educating others and initiated conversations around the importance of promoting awareness and early help-seeking for LC. Finally, having a large number of participants in each focus group increases the chances of some participants dominating the conversations. This risk was minimised through using icebreakers and having two experienced interviewers probe individuals who were not contributing to the discussions.

## Conclusion

Findings from this study suggest that promoting LC awareness, help-seeking, and ultimately early presentation and diagnosis can be achieved by developing and testing targeted interventions that appeal to at-risk populations and that take into account the format and characteristics of messages around LC. In the present study, participants favoured government-led multimodal (e.g. print and broadcast media) campaigns incorporating public health messages that are **S**imple, clear, honest; **W**orded positively; **I**ncorporating a shock element; **F**eaturing a celebrity, healthcare professional or cancer survivor; and **T**argeted **(SWIFT).**

## Supplementary Information

ESM 1 (DOCX 27 kb)

## Data Availability

All data have been reported in this study and supplementary tables.
