# Supplementary material for: Public Health Messaging and Strategies to Promote “SWIFT” Lung Cancer Detection: a Qualitative Study Among High-Risk Individuals
Source: J Cancer Educ. 2020 Oct 31;37(4):1026–35. doi: 10.1007/s13187-020-01916-w (PMC9399198; doi:10.1007/s13187-020-01916-w)
Supplement: Supplementary file 1 — (DOCX 27 kb) [file 13187_2020_1916_MOESM1_ESM.docx]

**Supplementary File**

**Table S1.** Semi-structured qualitative interview guide

| **Main questions** | **Sample probing questions** |
| --- | --- |
| What are your views and opinions regarding increasing the public’s awareness of the early signs and symptoms of lung cancer and encouraging them to seek help for these signs and symptoms? | - Is it important? Why? - What do you think about its risks and benefits? - What helps the public become more aware of the early signs and symptoms of lung cancer and to act on these signs and symptoms? (knowledge, skills, tools) |
| Think of your awareness and willingness to act on another health-related topic that you remember in detail, and is of interest to you: | - What is the topic? - How did you learn about it? - What drew your attention to it/ what makes it interesting? - Are you likely to act/ did you take any action based on what you learned from it? - Could the same method be used to raise awareness and get people to seek help for early signs and symptoms of lung cancer? If yes, how? If no, why not? |
| Are you personally interested in learning more about the early signs and symptoms of lung cancer and taking actions accordingly? | - Why? Can you please elaborate? - How confident are you in your ability to learn about the early signs and symptoms of lung cancer? - What would you like to know about lung cancer? - How would you like to learn about lung cancer? |
| As a nation, how do you think we can get people to seek help early for early signs and symptoms of lung cancer? | - What strategies can be used? - Why? |
| Participants were shown two distinct National Health Service (NHS) campaigns in the UK and were asked: | - How did you find the campaign? (format, colour, information, celebrity, personal stories) - Which of the two do you prefer? Why? - Can you see anything similar being done in Ireland? How? |

**Table S2.** Sample coding sheet from one of the focus groups

| **Context where excerpt was mentioned *(question/probe)*** | **Excerpt *(relevant direct quotes only, unedited)*** | **Code *(short statement capturing excerpt essence)*** |
| --- | --- | --- |
| **Raising awareness** | And I can’t understand why people are targeting older people. Why aren’t they targeting young people starting off?... If you got them young people, stop them. | **Target young people rather than older people** |
|  | Perhaps maybe by the doctors and that, if they can publish some facts that they can look at. | **Doctors can publish facts** |
|  | I was actually reading the paper on Sunday where somebody of 76, he’s a film star, a film actor or joining a band or something like that. He’s giving them up [cigarettes] at 76, even though he knows that his lungs are probably black inside, but he’s still giving them up at 76, so I said that’s great. I think he’s 76 and he’s giving them up, even though he knows at this stage that he probably has a lot of damage done. And I said to myself, well, sure, I’m a year younger than him. Maybe, you know. | **Read in the paper about a 76 y.o. celebrity who quit smoking despite damage**  **Thinks she can quit too** |
|  | I got into the habit of being up at seven in the morning, giving my husband his chemo. I’d go down, put on the kettle, wash my hands and have a cigarette, maybe two cigarettes and I’d watch the clock until eight o'clock to go up with his medication, the pain medication after that. And then at nine, I would feed him. He had to wait an hour to be fed after that and I’d have another few cigarettes. And I got into this habit of smoking… but now that he’s gone, I’m still sitting watching the clock at seven in the mornings. So I have to try and change my outlook on things. | **Smoking as part of routine that needs to be changed** |
|  | Absolutely 100% raise awareness, you know, in the schools and the young people. I do with my grandchildren, read books and I absolutely do everywhere I go and I’m not afraid to say that I tell them that I smoke and I wish I didn’t smoke. You know, I really do now. | **Pro- raising awareness in the schools among young people**  **Wishes she didn’t smoke** |
|  | Apart from the financial end of it, it’s the social end of it because you know, you’re going to meetings and things like that. You’re the only one smoking there, and then it draws back a lot of things you might like to do. There’s certain things I do now and I don't like because I smoke doing them, even though I’m very careful, I’m very clean and everything like that, but I’m kind of hiding that I smoke lately… because I’m probably embarrassed, ashamed, I don't know… I’m very particular about my habits in the house. One room only at the back for cigarettes. Anywhere else, no, and I’m on my own, but I’m very conscious of it. | **Embarrassment/shame/self-conscious due to smoking** |
|  | My mother never minded me smoking, but not in public was her method. I couldn’t go up the town for a walk, so I had to join the library in order to get out. | **Allowed to smoke but not in public** |
|  | I think if you get over a certain age, I think you should stay smoking | **Keep smoking if “over a certain age”** |
|  | I think myself a video should be done, a recording of some sort. You have a smoker that has cancer and then you have a survivor that had cancer and gave up the fags. And it would be good awareness of the difference of both and let the two sides tell the story. Why this person, even though they have cancer, why they can’t give up the fags or why they feel it’s not necessary to give them up or why they started smoking. The other person, why they gave them up, how long they were smoking before and what possessed them to start smoking in the first place. And the way that they can help others and like an alcoholic awareness thing. | **Video featuring story of a smoker that has cancer and a cancer survivor who quit smoking** |
|  | You can get support for it. Once they have a one-to-one support person that’s willing, that has given up the fags, knows exactly what. There’s no point in having somebody that never put a fag into their mouth to support you because they don’t know what you’re going through. You need somebody there that’s after being smoking, after giving them up, went through the mill, knows what you’re going through and is able to help you out there and then, you know. | **On-to-one support by someone who gave up smoking**  **No point having a never-smoker. Has to be someone who quit smoking** |
|  | In regards to the video, I think it’d be great to have the video of, two people, man, woman, whatever. Actually, one that has cancer and which way, which way they were feeling about it at the moment and which way it’s affecting their lives, you know what I mean… And then the non-smoker, that has been there, done that, knows exactly what the other person is going through because they’ve been there. So, there’s no looking down at one or another, oh, she smokes or this or that. They’re both on the same wavelength because they know what the other person is after going through, but unfortunately the person that had cancer doesn’t know what this other person went through to go off the fags, you know that sort of way because it is a big strong attempt for you to give up fags, especially, and depending how much he smoked in his life… It should be given out to teenagers. | **Video for teenagers featuring someone who smoked and someone who quit smoking** |
|  | I remember years ago, when I was a young fellow, I wasn’t in the school. Someone came into the school and showed a video of what lung cancer is, what cancer does to you. Now, a lot of lads I went to school with turned their back to it. I remember the day. They showed it in school and they never forgot it, never smoked. But I never saw it, you know what I mean. [Laughter]… Because we were only kids at the time. A lot of lads never forget what it was. I think it should be made compulsory, especially cancer, in children’s education. | **Recalling seeing a video at school about lung cancer. Lads turned their backs to it**  **Cancer should be made compulsory in children’s education** |
|  | But I think that’s too late for us… I smoked [cigarette brand name] all my life and I’ll tell you what, when I couldn’t afford them, I was smoking toilet paper… toilet paper, a bit of tobacco and then I started on the rollies. | **“It’s too late for us”**  **Could not afford cigarettes so smoked toilet paper then rollies** |
|  | I was trying it out with newspaper made into a fag when I was small, you know. I was only about 12 years of age. And I was caught and I got a slap across the ear and that was the end of that. But I never smoked then until I was 21 years of age again, do you know what I mean. And I remember my mother always said to me, ‘If you want to smoke, ask me. Don’t go behind my back and do it. Ask me’. And the first fag that I ever smoked then, I asked my mother for it and she used to smoke [cigarette brand name]. | **Caught smoking newspaper (12 y.o.) stopped then started again at 21. Mother gave her first cigarette** |
|  | My very first cigarette, I got it from a nun. A lovely nun. And she said to me, ‘There you are. You’ll be smoking for the rest of your life.’ And I am. | **Nun gave her first cigarette** |
|  | before I left my workplace today, there was a big meeting and part of the meeting was on a big screen, a video about cancer. Cancer awareness. And like there was 400 or something of us up on that site at [name of company]. So, everyone saw the video and it was, we were more tuned in than if there was just leaflets handed around…You might just throw them in your pocket or just flick through it…put it in the bin | **Before focus group, saw video about cancer at work. Video more effective than leaflets.** |
|  | You said how would you make young people aware? Give them proper information, truthful information. That’s the only way because young people are much more aware than what we ever were. When I was in secondary school, they showed this video of a man with a hole in his neck. | **Give young people proper truthful information**  **“Younger people are more aware than what we ever were”** |
|  | The pictures on the cigarette boxes. Horrible… guilt again, guilt. You feel guilty, you know… maybe I’m just sensitive. I don't know. Over the last couple of years now, I really want to be off them. | **Pictures of cigarette boxes horrible/leading to guilt and wanting to quit** |
|  | I think you look at them [pictures on cigarette boxes] for five minutes and then put the fag into the mouth, to be honest with you. | **Indifferent to pictures on cigarette boxes** |
|  | I know a man that buys, do you know the cigarette boxes? And he gets coloured paper and wraps them around the picture. | **Knowing someone who wraps cigarette boxes with coloured paper** |
|  | I think there’s a lot of peer pressure got to do with smoking and just like drugs. If you want to be in the gang, you go my way or no way… with the youngsters, it’s all peer pressure and if you want to be in a gang, most of them are smoking, so you’re going to smoke anyway. | **Like drugs, peer pressure plays a role in smoking among youngsters** |
|  | In regards to smoking, I think it’s a lot got to do with boredom as well. You get to a certain age, you’ve left work or whatever. When you have work, it sort of keeps you busy, keeps you going… empty hands and an empty mind… you’re looking for something to do. So they’re picking up a fag and they’re smoking it. | **Smoking related to boredom and not being busy/distracted by work** |
| **Scare tactics** | My oldest daughter, I don't know if you know this, I caught her smoking when she was seven. She used to smoke. And when I caught her, did she ever tell you?  I got her two hands, tied them behind her back. I put three cigarettes in her mouth. I lit the three of them. Tears were coming out of her ears. She has never smoked. | **Scare tactics to get daughter not to smoke** |
|  | My husband, many years ago, he was in hospital and the lady came around with a bit of a lung… A real lung and it frightened him so much, he never smoked again. | **Scare tactic: husband saw piece of lung and never smoked again** |
|  | My partner was in, it must be… I think it’s 10 years ago this month and he was a heavy smoker. He’d smoke maybe 40, 50 [cigarette brand name] a day. And he was driving trucks at the time. And tried his best to give them up and he couldn’t. He stayed smoking and he got really bad pains. His mother was after passing away. Everything kind of happened at once at Christmas and then he was feeling down in the dumps and he got pains here and there. So, I remember he went to the doctor. They sent him to [county name] and when he was down there for a few days, I went down to visit and the doctor called me in and he said, ‘I want to show you something.’ He showed me an X-ray of his lungs and one lung, they said he was at a 30% increased risk of lung cancer if he didn’t stop smoking there and then. And like he’s six foot three and a big bloke. If ever I saw a man quiver with fear, and he never smoked from that moment when the doctor showed me then. They said he’d be dead within six months. | **Partner who had pains quit smoking after seeing x-ray of his lungs and being told “he’d be dead within 6 months”** |
|  | If it was me, I would go to the schools and do you know these days out they get to the zoo? And they go down to [name of a park]. Bring them up to the hospice where the people are actually dying with cancer. Bring them on little tours to similar places. Show them what they’re like and you could be like that in 20 years, you know. You don’t smoke now, but just make sure you don’t do it. | **Scare tactics: Take children to hospices and show them people who are dying with cancer** |
|  | Blue sky with the clouds and that. The mammy up there going, ‘Bye bye, child.’ I spent all my life… | **Scare tactics: Mother (smoker) dying** |
| **Addiction** | Campaigning, I don't think it’s… Like all these videos are not working. But if you ask, do you have an addiction first of all? And that’s what you should be talking about, not smoking, not heroin. Actually, the addiction to nicotine, the withdrawal symptoms is worse than coming off of heroin. And yet they’ve rehabilitation centres for heroin addicts, but not for smokers. Why? Because there’s too many smokers, which is why they started all these campaigning to lower the amount of people smoking so that then they might have to use a health campaign. | **Video campaigning not working**  **Nicotine withdrawal symptoms are worse than coming off heroin**  **There are rehabilitation centres for heroin addicts but not for smokers**  **Campaigns help reduce the number of smokers** |
|  | We need help. If somebody chooses, I desperately need to stop smoking. Where’s the help? There is no help. | **Help to stop smoking is needed but not available** |
|  | I’ve tried the Nicorette patches. I’ve tried, remember the oil inhaler? Which I honestly, now, believe, this is the gospel truth, I almost got cancer from that because you used to slip it down your t-shirt. Did you ever try it? you just went… one puff. It’s not like these. They just contained a little sachet. And I got this red mark and the strange thing, but I ran to my doctor and I said, ‘I think I’ve cancer from this thing.’ I never got that from smoking. That’s the way, and I look, I know it affected my brain. My brain wants to go back. My body wants to go back on smoking. The addiction to nicotine. But you see, what they won’t release is there’s a hell of a lot more addictions because it says it on the Nicorette pack or pack when you get it. There’s rat poison. There’s… It’s not tar or nicotine is the problem. It’s everything else is in it and you’re also addicted to those in your system. | **Addicted to nicotine and all toxins**  **Tried Nicorette and oil inhaler**  **Was worried that oil inhaler, and not cigarettes, were giving her cancer** |
| **Ideal format** | A miracle | **Miracle to quit smoking** |
|  | If you even get the sniff that your daughter or your son is actually at a young age starting to take up smoking, this is the way you get them. You sit them down. You actually tell them, oh, so and so, especially if any of your family has died. You tell them what they died from, how much it killed them and how much it hurt them. | **Sit with own children and explain harms of smoking** |
|  | It might be through the television…advertise on telly. | **Advertise on TV** |
|  | Teach kids at a young age… and bring up through education. | **Teach kids at a young age** |
|  | Like I’ve a 17-year-old smoking vape fags. I mean they’re not good like. Why did they bring them shops? | **Vaping not good** |
|  | There is a change because I go around with work for the church here and I’m a lot of doors and it’s all outside smoking. You know, the ashtrays are outside. You wouldn't see that 10 years ago. Smo there is an awareness | **Indoor smoking ban caused more awareness** |
| **NHS Scotland campaign** | This one is very colourful. | **Colourful** |
|  | I’d be more drawn to this because there’s more… That, you seem to be drawn to that, to be honest with you [Laughter] Yes, you’re not really. | **More drawn to NHS Scotland campaign** |
|  | It has Alex Ferguson on it as well… Especially for young lads because they look up to him… It might be too late for me, but for my sons, they’d look up to him big time | **Celebrity important for young lads who look up to him** |
|  | I’d be more inclined to go for that one… Because of him [celebrity] | **Chooses NHS Scotland campaign because of celebrity** |
|  | Font | **Liked the font** |
|  | A picture says 100 words, 1,000 words… | **A picture says 100 words, 1,000 words** |
| **NHS England campaign** | This has, and the words are, you know what I mean. Circled off and there is more about it. It makes me aware of if I had a cough for more than three weeks to see my GP and don’t get scared, it says there, get checked. And I might go home and think about it. As I said, I avoid doctors as much as possible at the moment. Even to go for my prescription for my blood pressure now is a nightmare. | **Liked circled off words on NHS England campaign** |
|  | And you have scenarios…it raised my awareness of it. If I picked that up now and that, if I saw them in the doctors, I’d be more… I’d probably flick through the two of them, but I’d be more drawn to this because of these and because of the…[scenarios] | **Two personal stories/scenarios raised awareness** |
|  | I’d probably look at that. I know the colours on that. I tend to go for this. Like I say, if I flick through the two of them and I’d seen all these | **Drawn to colour** |
